# Lymphogranuloma venereum presenting as a giant rectal ulcer with inguinal lymphadenitis: a case report

**DOI:** 10.1093/jscr/rjag899

**Published:** 2026-10-07

**Authors:** Yidian Lei, Xiufeng Zhang, Zhong Shen, Zhiyong Liu, Mengjie Zhong

**Affiliations:** Department of Colorectal Surgery, The Hangzhou Third People’s Hospital, No. 38 Westlake Avenue, Hangzhou City, Zhejiang Province 310009, China; Department of Colorectal Surgery, The Hangzhou Third People’s Hospital, No. 38 Westlake Avenue, Hangzhou City, Zhejiang Province 310009, China; Department of Colorectal Surgery, The Hangzhou Third People’s Hospital, No. 38 Westlake Avenue, Hangzhou City, Zhejiang Province 310009, China; Department of Colorectal Surgery, The Hangzhou Third People’s Hospital, No. 38 Westlake Avenue, Hangzhou City, Zhejiang Province 310009, China; Department of Colorectal Surgery, The Hangzhou Third People’s Hospital, No. 38 Westlake Avenue, Hangzhou City, Zhejiang Province 310009, China

**Keywords:** lymphogranuloma venereum, rectal ulcer, anorectal proctitis, men who have sex with men, rectal malignancy mimic, doxycycline

## Abstract

Anorectal lymphogranuloma venereum (LGV) can mimic rectal malignancy, but diagnosis may be challenging when LGV-specific serovar testing is unavailable. A 26-year-old man who has sex with men presented with hematochezia and increased bowel movement frequency. Colonoscopy revealed a large deep rectal ulcer with mucopurulent exudate, and pelvic magnetic resonance imaging showed rectal wall thickening with regional lymphadenopathy, raising concern for rectal cancer. Histopathology showed inflammatory changes without malignancy. Rectal secretion testing was positive for *Chlamydia trachomatis* RNA, and the patient was clinically diagnosed with probable anorectal LGV. Oral doxycycline 100 mg twice daily for 21 days led to marked ulcer regression at 3 weeks and near-complete mucosal healing by 6 weeks. This case highlights the importance of considering sexually transmitted proctitis in young men who have sex with men patients with atypical rectal ulcers.

## Introduction

*Chlamydia trachomatis* is a common sexually transmitted intracellular pathogen [1, 2]. Lymphogranuloma venereum (LGV), caused by invasive *C. trachomatis* serovars L1–L3, may present as severe proctitis, rectal ulceration, and lymphadenitis in men who have sex with men (MSM) [3–5]. Because its endoscopic and radiological findings can resemble inflammatory bowel disease or rectal malignancy, delayed diagnosis may lead to unnecessary invasive treatment [3, 6, 7]. We report a probable anorectal LGV case presenting as a giant rectal ulcer with inguinal lymphadenitis.

## Case presentation

A 26-year-old MSM was admitted with a 1-month history of hematochezia and increased bowel movement frequency after recent same-sex intercourse. He had low-grade fever, slightly elevated inflammatory markers, enlarged inguinal lymph nodes, and rectal wall thickening ~3 cm from the anal verge on digital rectal examination. Tests for syphilis, human immunodeficiency virus (HIV), *Mycobacterium tuberculosis*-specific cellular responses, and *Treponema pallidum*-specific staining were negative.

Pelvic magnetic resonance imaging (MRI) showed diffuse lower rectal wall thickening with multiple surrounding enlarged lymph nodes, initially raising concern for rectal malignancy (Fig. 1a). Colonoscopy revealed extensive mucosal erosion, inflammatory hyperplasia, mucopurulent exudate, and a large deep ulcer involving about half of the rectal circumference (Fig. 1b). However, the lesion appeared predominantly inflammatory rather than mass-forming. Rectal biopsy showed inflammatory cell infiltration, necrotic exudate, and histiocytic hyperplasia, without malignant epithelial cells, dysplasia, or tumor-forming architecture. Typical features of inflammatory bowel disease, such as chronic crypt architectural distortion, basal plasmacytosis, or diagnostic granulomas, were absent. Left inguinal lymph node biopsy demonstrated chronic granulomatous inflammation with focal suppuration, favoring infectious lymphadenitis over metastatic disease.

**Figure 1:**
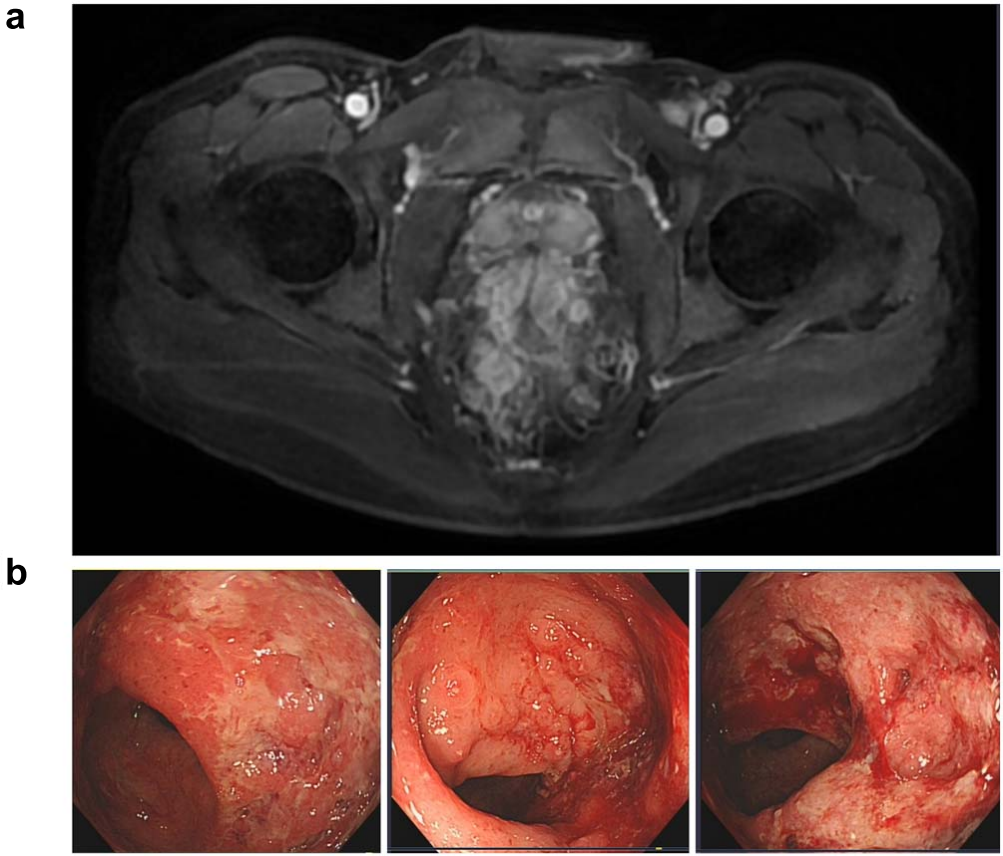
Pre-treatment imaging and endoscopic findings. (a) Pelvic MRI. Axial T2-weighted imaging demonstrates diffuse wall thickening in the lower rectum with multiple surrounding enlarged lymph nodes, initially raising concern for rectal malignancy. (b) Endoscopic appearance at initial presentation. The lower rectum exhibits extensive mucosal erosion, inflammatory hyperplasia, and a large, deep ulcer covering approximately half of the intestinal circumference, with mucopurulent exudate, characteristic of highly invasive features.

Rectal secretions were positive for *Ureaplasma urealyticum* and *C. trachomatis* RNA. LGV-specific L1–L3 serovar testing was unavailable; therefore, a clinical diagnosis of probable anorectal LGV was made based on MSM history, severe ulcerative proctitis, inguinal lymphadenopathy, positive rectal *C. trachomatis* RNA, exclusion of malignancy and common alternative causes, and subsequent treatment response [7–9]. Oral doxycycline 100 mg twice daily was administered for 21 days. Colonoscopy after 3 weeks showed marked ulcer regression (Fig. 2a), and near-complete mucosal healing was observed after 6 weeks (Fig. 2b). Two months after treatment, rectal secretion tests for *U. urealyticum* and *C. trachomatis* RNA were negative.

**Figure 2:**
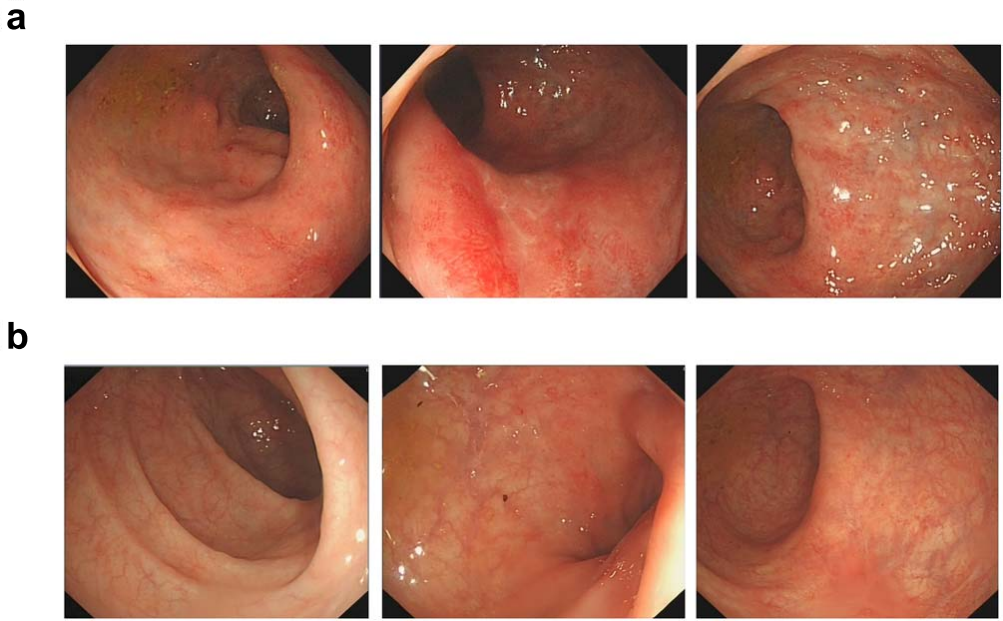
Comparison of endoscopic follow-up findings after treatment. (a) Follow-up colonoscopy after 3 weeks of doxycycline treatment. The rectal mucosal ulcer is significantly reduced in size, with decreased exudate; mucosal congestion and inflammatory edema are markedly improved compared to baseline, indicating a favorable therapeutic response. (b) Final follow-up colonoscopy after 6 weeks of treatment. The results show complete healing of the rectal mucosa and resolution of the ulcer; vascular patterns are clearly restored, with no evidence of intestinal stricture or scarring.

## Discussion

This case emphasizes that sexually transmitted proctitis should be considered in young MSM patients with atypical rectal ulcers. Rectal wall thickening, hematochezia, a large ulcer, and lymphadenopathy can mimic rectal cancer, and previous reports have described LGV presenting as ulcerative or mass-like rectal lesions [6, 7]. Nevertheless, diffuse inflammatory mucosal changes, absence of a discrete tumor, negative histopathology for carcinoma, and response to doxycycline favored infection in this patient.

The diagnosis should be interpreted as probable rather than microbiologically confirmed LGV because LGV-specific serovar identification was not performed. Ideally, *C. trachomatis* nucleic acid amplification testing should be followed by LGV-discriminatory molecular typing [5, 8]. In clinical practice, probable LGV may be diagnosed when severe anorectal symptoms, inguinal lymphadenopathy, positive rectal *C. trachomatis* testing, and epidemiological risk factors are present. The recommended treatment is doxycycline 100 mg twice daily for 21 days [5, 9].

Differential diagnosis includes non-LGV anorectal chlamydial infection, gonococcal proctitis, syphilitic proctitis, herpes simplex virus proctitis, cytomegalovirus proctitis, amebic colitis, tuberculosis, inflammatory bowel disease, and rectal cancer [9]. Inguinal lymphadenopathy was an important clue: although it may be misinterpreted as metastatic disease, lymphatic involvement, and lymphadenitis are characteristic of LGV [4, 5]. Thus, enlarged inguinal nodes in a sexually active MSM patient should prompt sexual history-taking and targeted STI testing.

The concomitant *U. urealyticum* positivity should be interpreted cautiously because it may represent colonization [10]. Its role in causing a giant rectal ulcer is uncertain, and the overall clinicopathological picture was more consistent with *C. trachomatis*-associated proctitis/probable LGV. Because doxycycline is active against some Ureaplasma infections, post-treatment negativity does not prove that *U. urealyticum* was the primary pathogen.

## Conclusion

Probable anorectal LGV can mimic rectal cancer or inflammatory bowel disease. In young MSM patients with giant rectal ulcers, rectal wall thickening, and inguinal lymphadenopathy, early rectal *C. trachomatis* testing and clinicopathological correlation are essential to avoid misdiagnosis and unnecessary surgery.

## Data Availability

The data involved in this study are available from the corresponding author upon reasonable request.
